# Threshold heterogeneity of perioperative hemoglobin drop for acute kidney injury after noncardiac surgery: a propensity score weighting analysis

**DOI:** 10.1186/s12882-022-02834-3

**Published:** 2022-06-11

**Authors:** Yan Zhou, Si Liu

**Affiliations:** 1 Department of Anesthesiology and Critical Care Medicine, Peking University First Hospital, Beijing, 100034 China; 2 Department of Database Center, Peking University First Hospital, Beijing, 100034 China

**Keywords:** Acute kidney injury, Creatinine, hemoglobin drop, Minimum *P*-value, Noncardiac surgery, Propensity score weighting

## Abstract

**Background:**

Perioperative hemoglobin drop after noncardiac surgery is associated with acute kidney injury (AKI). However, opinion on the tolerable difference in postoperative hemoglobin drop in patients with different preoperative hemoglobin levels does not reach a consensus. This study aimed to identify hemoglobin drop thresholds for AKI after noncardiac surgery stratified by preoperative hemoglobin levels.

**Method:**

This was a single-center retrospective cohort study for elective noncardiac surgery from January 1, 2012, to December 31, 2018. The endpoint was the occurrence of AKI 7 days postoperatively in the hospital. The generalized additive model described the non-linear relationship between hemoglobin drop and AKI occurrence. The minimum *P*-value approach identified cut-off points of hemoglobin drop within postoperative 7 days for patients with or without preoperative anemia. Stratified by preoperative anemia, hemoglobin drop’s odds ratio as continuous, quintile and dichotomous variables by various cut-off points for postoperative AKI were calculated in multivariate logistic regression models before and after propensity score weighting (PSW).

**Results:**

Of the 35,631 surgery, 5.9% (2105 cases) suffered postoperative AKI. Non-linearity was found between hemoglobin drop and postoperative AKI occurrence. The thresholds and corresponding odds ratio of perioperative hemoglobin drop for patients with and without preoperative anemia were 18 g/L (1.38 (95%CI 1.14 -1.62), *P* < .001; after PSW: 1.42 (95%CI 1.17 -1.74), *P* < .001) and 43 g/L (1.81 (95%CI 1.35—2.27), *P* < .001; after PSW: 2.88 (95%CI 1.85—4.50), *P* < .001) respectively. Overall thresholds and corresponding odds ratio were 43 g/L (1.82 (95%CI 1.42—2.21)), *P* < .001; after PSW: 3.29 (95%CI 2.00—5.40), *P* < .001). Sensitivity analysis showed similar results. Heterogeneity subgroup analysis showed that intraoperatively female patients undergoing intraperitoneal surgery without colloid infusion seemed to be more vulnerable to higher hemoglobin drop. Further analysis showed a possible linear relationship between preoperative hemoglobin and perioperative hemoglobin drop thresholds. Additionally, this study found that the creatinine level changed simultaneously with hemoglobin level within five postoperative days.

**Conclusions:**

Heterogeneity of hemoglobin drop endurability exists after noncardiac non-kidney surgery. More care and earlier intervention should be put on patients with preoperative anemia.

**Supplementary Information:**

The online version contains supplementary material available at 10.1186/s12882-022-02834-3.

## Background

Perioperative hemoglobin drop in noncardiac surgery is related to postoperative kidney injury. Preoperative hemoglobin level is a critical risk factor for postoperative AKI. There is no recognized tolerance threshold for postoperative hemoglobin reduction among different preoperative hemoglobin levels. These parameters are essential for the prevention of perioperative renal injury. We assume that hemoglobin drop within 7 days after the operation will affect perioperative AKI and that patients with different preoperative hemoglobin statuses have different tolerance thresholds. Based on this hypothesis, this retrospective observational study aimed to identify the hemoglobin drop threshold of patients with different preoperative hemoglobin levels. Moreover, trying to understand the relationship between hemoglobin reduction and blood creatinine level within 7 days after surgery.

## Methods

### Study design, setting, population, and data collection

This retrospective observational cohort study was undertaken at Peking University First Hospital in China, a 1500-bed teaching hospital. The Ethics Committee approved this study.

Due to the study’s retrospective nature, the lack of patient follow-up, and the absence of patient identification information, the IRB waived the necessity for written informed consent.

This study extracted data from the hospital’s perioperative database [1]. Adult (age ≥ 18 years old) who underwent elective noncardiac non-kidney surgery between January 1, 2012, and December 31, 2018, were screened. Surgery identification was based on the International Classification of Diseases and Procedures, Ninth Clinical Revision volume 3 (ICD-9-v3).

The procedures included otolaryngology, general surgery, urology, gynecology, orthopedics, neurosurgery, and vascular and thoracic surgery, excluding cardiac, kidney, obstetrics, and emergency surgery. Only the first procedure record was used by patients who have been operated on more than two times a year. If the two operations’ time intervals were less than three months, the second operation would not be registered. Surgery under local infiltration anesthesia was also omitted from this analysis.

### Variables used in the present study

#### Hemoglobin drop

All preoperative hemoglobin results 3 months before surgery were collected in the dataset. Within these results, the value closest to the surgery date was used. For postoperative anemia, only results 7 days after surgery were collected.

Postoperative hemoglobin value was collected 7 days after surgery day by day. Sometimes several HGB tests were performed within one day, and only the lowest was defined as the minimum HGB. Furthermore, hemoglobin drop (HGBd) was calculated using the preoperative value minus each of the minimum HGB values after surgery; in this case, we would get 7 values of minimum hemoglobin level and hemoglobin drop. Likewise, creatinine was calculated similarly, with the name creatinine level and creatinine increment (each max postoperative creatinine minus preoperative creatinine).

#### Preoperative anemia

In this study, preoperative hemoglobin levels less than 120 g/L in females or 130 g/L in male patients were defined as preoperative anemia. The hemoglobin was tested closest to the date of surgery.

The information on each patient collected in the present study included their demographic and essential characteristics (e.g., body mass index, BMI, gender, age, smoking, and alcohol habits); preoperative co-existing disorders, and lab tests (e.g., hypertension, coronary artery disease, preoperative hemoglobin, albumin, creatinine levels), and intraoperative parameters (e.g., intraoperative hypotension, blood transfusion, anesthetic technique, The modified John Hopkins Hospital criteria, MJHSC, whether or not intraperitoneal surgery, mean intraoperative heart rate)0.10,11 The modified John Hopkins Hospital criteria (MJHSC) were used to categorize the surgical complexity [10, 11]. (Variables definition was shown in Table S1).

#### Stratification of the dataset

This study stratified the dataset into patients with or without preoperative anemia. We defined preoperative anemia criteria: Hemoglobin value preoperatively less than 130 g/L for male patients and 120 g/L for female patients.

### Study endpoints

The primary endpoint was any patient with AKI within seven days after surgery in the hospital. The authors used KDIGO as the criteria for AKI, defined by the patient’s postoperative serum creatinine level increase to no less than 26.5 μmol/l within 48 h, or 1.5 times from the baseline 7 days after surgery, or initialization of blood dialysis [2–8]. GFR value or urine output was not included in the definition of the outcome in this study, as postoperative creatinine level dramatically fluctuates, which could cause an inaccurate estimate of eGFR, and urine output was not accurately measured by volume or weight in routine practice. A portion of patients was not with a urinary catheter during the operation.

### Statistical analysis

Patients were separated into two groups according to the hemoglobin drop cut-off point. Continuous variables with a normal or non-normal distribution were compared using the Student t-test or Mann–Whitney U-test. The Kolmogorov–Smirnov test was used to determine whether the data were normally distributed. Categorical variables were compared using the Chi-Square test or the continuity corrected Chi-Square test. Rank variables were compared using the Kruskal–Wallis H-test. A two-sided *p*-value < 0.05 was considered significant.

### Non-linear relationship between hemoglobin drop and AKI

This study examined the hemoglobin drop’s unadjusted and fully adjusted relationship and AKI risk using a restricted cubic spline function by Generalized Additive Models (GAM). The marginal effect of preoperative hemoglobin drop on postoperative AKI was calculated and plotted.

### Minimum *P*-value approach for hemoglobin drop threshold

This study tried to locate the inflection point dividing the hemoglobin drop into two clinically meaningful categories [9–13]. If we observed an inflation area, the hemoglobin drop’s optimal threshold was determined using the minimum *P*-value approach. This approach evaluated every possible threshold of the hemoglobin drop at intervals of 1 g/L in the multivariate logistic regression models. The hemoglobin drop that demonstrated the smallest statistically significant *P*-value was selected as the optimal threshold to divide the hemoglobin drop into two groups. This approach was used for the whole dataset and stratified subsets of patients with or without preoperative anemia.

### Heterogeneity of hemoglobin drop threshold

To better illustrate the relationship between preoperative hemoglobin level and perioperative hemoglobin drop endurance, the whole dataset was divided into multiple mini-datasets; every mini-dataset had a preoperative hemoglobin span of 3 g/L. (for instance, 100 g/L -103 g/L, 101 g/L -104 g/L,102 g/L -105 g/L …… from 90 to 160 g/L). The threshold for every mini-dataset was calculated by the same approach stated above. A plot with these thresholds and corresponding preoperative hemoglobin from 90 to 160 g/L was illustrated.

### Multivariate logistic regression to detect the association between hemoglobin drop and AKI

This study created crude, age, and sex-adjusted, fully adjusted multivariate logistic models, and confounders were assessed based on a priori knowledge and other studies [2–8, 14–39]. The following variables were considered: gender, age, BMI, preoperative hypertension, preoperative hemoglobin, creatinine and albumin levels, surgical complexity (MJHSC [11]), whether or not intraperitoneal and cancer surgery, intraoperative hypotension, blood transfusion, dexmedetomidine, and colloid infusion. Variance inflation factor with a reference value of 2 was set for multicollinearity consideration. Hosmer–Lemeshow test was used for the goodness of fit [40]. This study assessed whether the addition of the hemoglobin drop to the model could improve the predictive ability for AKI by calculating the category-free net reclassification improvement (NRI), the integrated discrimination improvement (IDI), and c-statistic. The integrated discrimination improvement (IDI) index is a good tool for evaluating the capacity of a marker to predict a binary outcome of interest.

## Heterogeneity analysis

A heterogeneity analysis was conducted to evaluate any differences in the treatments’ effects among different subgroups by covariates included in the model. The adjusted odds ratio for AKI in each subgroup was calculated, and the interaction effect of covariates was tested. Sensitivity logistic regression models were constructed as follows: (i) exclusion of patients with Intraoperative hypotension; (ii) with surgery duration adjustment; (iii) preoperative hemoglobin mean level within three months instead of the hemoglobin value tested closest to the date of surgery; (iv) 1:1 propensity score matching analysis.

### Analysis of the propensity score weighting (PSW)

To increase the robustness, the present study balanced the two groups (patients’ hemoglobin drop below or above the cut-off point) using the propensity score weighting (PSW), which diminishes the effects of measured confounding factors and obtains a less biased result in observational studies. The PSW was calculated using gradient boosted regression models, in which whether patients’ hemoglobin drop was below or above the cut-off point was the dependent variable [12, 13]. Unbalanced preoperative factors (age, gender, body mass index, smoking and drinking habits, preoperative hemoglobin, albumin, creatinine level, co-existing disorders, surgery duration, cancer surgery, intraoperative blood transfusion, surgical complexity, intraperitoneal, anesthesiology experience) was included as independent variables. Hemoglobin drop’s adjusted odds ratio from two new cohorts with propensity weights was calculated and considered robust and less biased. This study calculated PSW results for the whole dataset and sub-dataset stratified by preoperative anemia.

### Statistical packages

Data management, statistical analysis and plot drawing were performed using the R programming language (v.3.5.2).

## Result

**Fig. 1:**
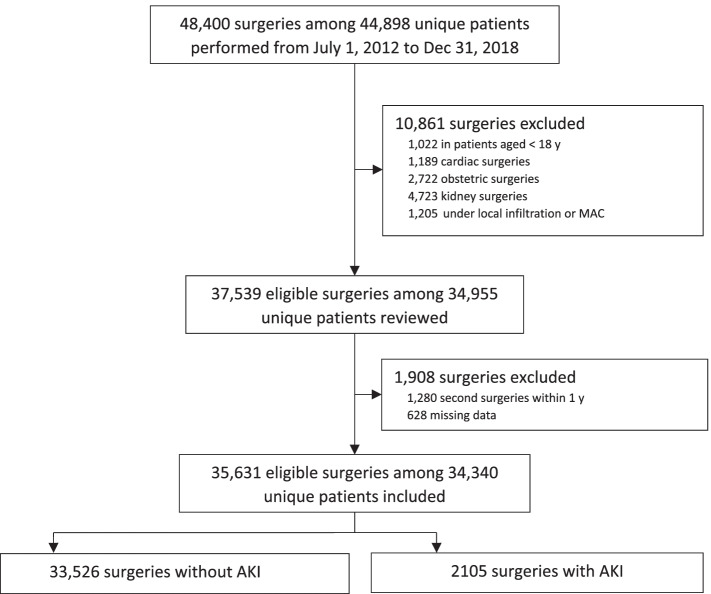
Flow diagram of the study population. AKI: acute kidney injury

For the period March 2012 to December 2018. 48,400 elective noncardiac surgery were screened, and ultimately a total of 35,631 surgery were analyzed (Fig. 1).

The median age was 61 years old, 54.9% male and the mean body mass index was 24.5 ± 3.8 kg/m^2^, mostly ASA class II and III. The most common surgery was the digestive tract (39.4%) and Genital/urinary surgery (24.0%), with a median operation duration of 130 (74–214 interquartile range, IQR) minutes, and the median hemoglobin drop was 13 (from 6 to 22) g/L (Table 1).

**Table 1:** Patient characteristics and operative variables stratified by hemoglobin drop

**Characteristic**	**ALL (*n* = 35,631)**	**HGBd** ≤ **43 (*n* = 34,479)**	**HGBd > 43 (*n* = 1152)**	***P* value**
**Age** [yr; median (IQR)]	61(49–71)	61(49–71)	60(49–68)	< .001
**Male gender**, [n (%)]	19,570(54.9%)	18,843(54.7%)	727(63.1%)	< .001
**Smoking**	5297 (14.9%)	5099 (14.8%)	198 (17.2%)	0.024
**Drinking**	4538 (12.7%)	4348 (12.6%)	190 (16.5%)	< .001
**Body mass index**, [mean (SD)] kg/m^2^	24.5 ± 3.8	24.5 ± 3.8	23.8 ± 3.6	< .001
**Co-existing disease**				
hypertension	13,243(37.2%)	12,851(37.3%)	392(34.0%)	0.027
Coronary artery disease	5865(16.5%)	5702(16.5%)	163(14.1%)	0.035
Heart failure	539(1.5%)	512(1.5%)	27(2.3%)	0.026
Arrhythmia	1244(3.5%)	1208(3.5%)	36(3.1%)	0.544
Peripheral arterial disease	681(1.9%)	665(1.9%)	16(1.4%)	0.227
Stroke	3501(9.8%)	3409(9.9%)	92(8.0%)	0.037
diabetes mellitus	5128 (14.4%)	4993.0 (14.5%)	135.0 (11.7%)	0.009
**Preoperative serum creatinine**, [mean (SD)] mmol/L	81.1 (19.3)	81.0 (19.3)	83.2 (19.3)	< .001
**Preoperative serum albumin**, [mean (SD)] g/L	41.0 (5.9)	41.0 (5.9)	41.8 (6.2)	< .001
**Preoperative hemoglobin**, [median (IQR)] g/L	136(123–148)	136(123–147)	144(134–153)	< .001
**Postoperative hemoglobin**, [median (IQR)] g/L	128(116–140)	128(116–140)	121(109–134)	< .001
**rCRI**				
0	30,302(85.0%)	29,299(85.0%)	1003(87.1%)	0.030
1	3232(9.1%)	3129(9.1%)	103(8.9%)	
2	1465(4.1%)	1429(4.1%)	36(3.1%)	
≥ 3	632(1.8%)	622(1.8%)	10(0.9%)	
**ASA**				
I	5841 (16.4%)	5706 (16.5%)	135 (11.7%)	< .001
II	25,639 (72.0%)	24,798 (71.9%)	841 (73.0%)	
III	3986 (11.2%)	3832 (11.1%)	154 (13.4%)	
IV or V	165 (0.5%)	143 (0.4%)	22 (1.9%)	
**Surgery type**				
Eye/ear/throat	281(0.8%)	277(0.8%)	4(0.3%)	< .001
Integumentary	321(0.9%)	311(0.9%)	10(0.9%)	
Genital/urinary	8567(24.0%)	8376(24.3%)	191(16.6%)	
Musculoskeletal	4477(12.6%)	4330(12.6%)	147(12.8%)	
Nervous	2536(7.1%)	2390(6.9%)	146(12.7%)	
Vascular	760(2.1%)	728(2.1%)	32(2.8%)	
Digestive	14,022(39.4%)	13,495(39.1%)	527(45.7%)	
Respiratory	3423(9.6%)	3357(9.7%)	66(5.7%)	
Other	1244(3.5%)	1215(3.5%)	29(2.5%)	
**Surgery time**, [min; median (IQR)]	130(74–214)	127(73–209)	254(152–347)	< .001
**Anesthesia duration**, [min; median (IQR)]	208(140–308)	204(139–301)	369(262–473)	< .001
**Anesthesia type**				
General anesthesia	31,096(87.3%)	30,003(87.0%)	1093(94.9%)	< .001
General anesthesia + epidural/nerve block	4535(12.7%)	4476(13.0%)	59(5.1%)	
**Intraoperative fluid administration** [median (IQR)]				
Infusion volume	1600(1100–2450)	1600(1100–2350)	4000(2600–5700)	< .001
Crystal	1300(1000–1950)	1300(1000–1850)	2600(1700–3500)	< .001
Colloid	0(0–500)	0(0–500)	1000(500–1500)	< .001
**Estimated blood loss**, [ml; median (IQR)]	50(0–200)	30(0–200)	600(200–1400)	< .001
**Intraoperative blood infusion**	5720(16.1%)	5078(14.7%)	642(55.7%)	< .001
**Intraoperative blood salvage**	1767(5.0%)	1582(4.6%)	185(16.1%)	< .001
**Urine**, [ml; median (IQR)]	200(0–500)	200(0–500)	550(200–1000)	< .001
**Intraoperative hypotension**	8541(24.0%)	8133(23.6%)	408(35.4%)	< .001
**Intraoperative mean HR**, bpm				
60–65	7517(21.4%)	7303(21.5%)	214(18.7%)	< .001
< 60	9721(27.7%)	9515(28.0%)	206(18.0%)	
65–75	10,364(29.5%)	9990(29.4%)	374(32.6%)	
> 75	7485(21.3%)	7132(21.0%)	353(30.8%)	

a: All values are reported as No. (%) unless otherwise specified

*HGBd* Hemoglobin drop, the difference between preoperative mean hemoglobin and perioperative minimal hemoglobin, g/L

*rCRI* revised Cardiac Risk Index

*IQR* Interquartile

Of the 35,631 surgery, 2105 (5.9%) developed AKI, and most of them occurred on the first day after surgery; Patients with higher hemoglobin drops had a more extended hospitalization (8 vs. 5 days; *P* < 0.001). The logistic regression showed that besides old age, extreme BMI, low preoperative albumin or hemoglobin level, Intraperitoneal surgery, cancer or major surgery, intraoperative hypotension, and colloid infusion were all risk factors, while intraoperative dexmedetomidine was a protective factor (Table 2).

**Table 2:** Models to predict postoperative AKI; aOR, adjusted odds ratio; CI, confidence interval;

	**Patient/operative variables only**		**Patient/operative variables and HGBd**	
	**aOR (95% CI)**	***P*-value**	**aOR (95% CI)**	***P*-value**
**Gender**, (female)	1.32 (1.18—1.45)	< .001	1.33 (1.19—1.46)	< .001
**Age**, (< 40 yr)	reference	< .001	reference	< .001
40—50	1.44 (1.13—1.75)		1.45 (1.14—1.77)	
50—60	1.53 (1.22—1.84)		1.55 (1.24—1.86)	
60—70	1.32 (1.06—1.59)		1.34 (1.07—1.60)	
> 70	1.86 (1.50—2.23)		1.88 (1.51—2.24)	
**Body mass index**, (18.5–24.9 kg/m^2^)	reference	< .001	reference	< .001
< 18.5	1.46 (1.17—1.75)		1.45 (1.16—1.74)	
25.0–29.9	1.12 (1.00—1.24)		1.13 (1.00—1.25)	
> 30.0	1.29 (1.12—1.47)		1.32 (1.14—1.49)	
**Hypertension**	1.00 (0.86—1.14)	0.992	1.01 (0.87—1.15)	0.909
**Preoperative hemoglobin level,** 130 – 140 g/L	reference	< .001	reference	< .001
< 110	2.02 (1.71—2.34)		2.11 (1.78—2.44)	
110—120	1.25 (1.02—1.47)		1.27 (1.04—1.50)	
120—130	0.98 (0.82—1.15)		0.99 (0.83—1.16)	
> 140	1.26 (1.09—1.44)		1.24 (1.07—1.41)	
**Preoperative albumin level,** > 40 mg/L	1.76 (1.58—1.94)	< .001	1.77 (1.59—1.95)	< .001
**Cancer to benign surgery**	1.45 (1.30—1.59)	< .001	1.43 (1.28—1.57)	< .001
**Intraperitoneal surgery**	0.74 (0.66—0.83)	< .001	0.74 (0.66—0.83)	< .001
**Intraoperative hypotension**	1.72 (1.46—1.97)	< .001	1.69 (1.45—1.94)	< .001
**Intraoperative blood transfusion**	0.98 (0.86—1.09)	0.684	0.92 (0.81—1.03)	0.173
**Intraoperative dexmedetomidine use**	0.81 (0.73—0.89)	< .001	0.81 (0.73—0.89)	< .001
**Intraoperative colloid use**	1.21 (1.09—1.33)	< .001	1.18 (1.06—1.30)	0.001
**Hemoglobin drop**, (≤ 43 g/L)	reference	-	reference	< .001
> 43	-		1.82 (1.42—2.21)	

*GA* General Anesthesia

*HGBd* Hemoglobin drop, the difference between preoperative mean hemoglobin and perioperative minimal hemoglobin, g/L

*CI* Confidence Interval

*aOR* adjusted Odds Ratio

### Non-linear relationship detection between hemoglobin drop and AKI

The restricted cubic spline function in the crude and adjusted GAM models describing the hemoglobin drop to AKI was a "J" shaped curve, with an inflection point at approximately 40 g/L, after which the probability of AKI rose straight up (Fig. S1).

### Hemoglobin drop thresholds heterogeneity

The minimal *P*-value approach showed that 43 g/L of hemoglobin drop was the whole dataset’s cut-off point. After stratification, the perioperative hemoglobin drop thresholds were 18 and 43 g/L for patients with and without preoperative anemia.

**Fig. 2:**
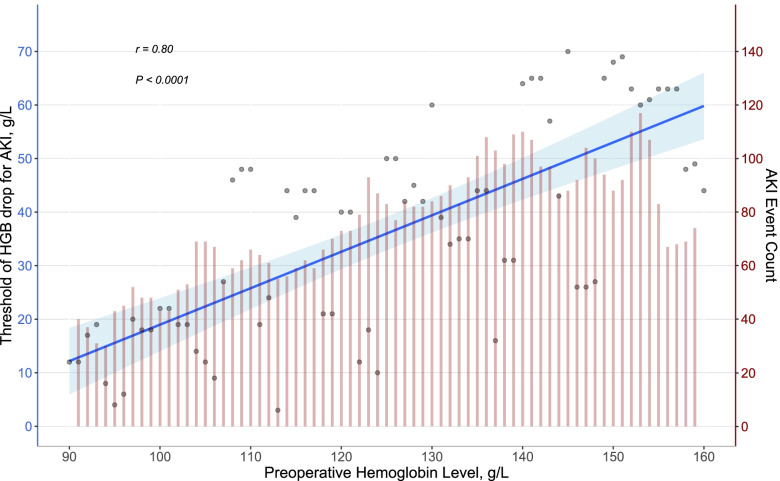
Continuous hemoglobin drop thresholds for multi-mini-datasets with preoperative hemoglobin. The whole dataset was divided into multiple mini-datasets. Every mini-dataset had a preoperative hemoglobin span equaled to 3 g/L. (for instance, 100 g/L -103 g/L, 101 g/L -104 g/L,102 g/L -105 g/L …… from 90 to 160 g/L). The threshold for every mini-dataset was calculated and presented as black dots. A linear regression line was plotted, shaded areas represent 95% confidence intervals. With the preoperative hemoglobin level decrease, the thresholds decrease dramatically accordingly. AKI: acute kidney injury

Hemoglobin drop thresholds for multi-mini-datasets with preoperative hemoglobin (span of 3 g/L) from 90 to 160 g/L were demonstrated in Fig. 2. With the preoperative hemoglobin level decreasing, the thresholds dramatically decrease accordingly (Fig. 2).

### Logistic regression for detecting any association between hemoglobin drop and AKI

The multivariate logistic regression results showed an association between hemoglobin drop and postoperative AKI. The fully adjusted odds ratio for hemoglobin drop cut-off point (hemoglobin drop < 43 g/L as reference), was 1.82 (1.42—2.21), *P* < 0.001 (Table 3).

**Table 3:** AKI’s Odds ratio for hemoglobin drop as a continuous variable, as a categorical variable by IQR or cut-point in crude and adjusted models in total and stratified datasets

	**Model 1**		**Model 2**		**Full model**	
	**AKI aOR (95% CI)**	***P*-value**	**AKI aOR (95% CI)**	***P*-value**	**AKI aOR (95% CI)**	***P*-value**
**Full dataset**						
**Continous HGBd**	1.01 (1.01—1.01)	< .001	1.01 (1.01—1.01)	< .001	1.02 (1.01—1.02)	< .001
**HGBd quintile, g/L**						
< 4	reference		reference		reference	
4—10	1.20 (1.03—1.38)	0.012	1.16 (1.00—1.33)	0.039	1.31 (1.12—1.51)	< .001
10—17	1.16 (1.00—1.32)	0.031	1.14 (0.98—1.30)	0.06	1.40 (1.20—1.60)	< .001
17—25	1.26 (1.08—1.44)	0.001	1.21 (1.04—1.38)	0.008	1.57 (1.33—1.80)	< .001
> 25	1.33 (1.14—1.51)	< .001	1.26 (1.08—1.44)	0.001	1.65 (1.38—1.92)	< .001
**HGBd cut-point before PSW**	reference	< .001	reference	< .001	reference	< .001
≤ 43						
> 43	1.77 (1.42—2.13)		1.80 (1.44—2.17)		1.82 (1.42—2.21)	
**HGBd cut-point after PSW**	reference	< .001	reference	< .001	reference	< .001
≤ 43						
> 43	2.66 (1.78—3.97)		2.68 (1.79—4.02)		3.29 (2.00—5.40)	
**Patient with preoperative anemia**						
**Continous HGBd**	1.02 (1.01—1.02	< .001	1.02 (1.01—1.02	< .001	1.01 (1.01—1.02)	< .001
**HGBd quintile, mmHg**						
< 0	reference		reference		reference	
0—6	1.26 (0.97—1.55)	0.053	1.15 (0.88—1.42)	0.238	1.15 (0.88—1.43)	0.247
6—12	1.24 (0.96—1.52)	0.064	1.13 (0.87—1.39)	0.293	1.12 (0.86—1.38)	0.343
12—20	1.26 (0.97—1.54)	0.049	1.15 (0.88—1.41)	0.244	1.14 (0.87—1.40)	0.278
> 20	1.73 (1.35—2.12)	< .001	1.64 (1.27—2.00)	< .001	1.50 (1.14—1.86)	0.001
**HGBd cut-point before PSW**	reference	< .001	reference	< .001	reference	< .001
≤ 18						
> 18	1.48 (1.24—1.71)		1.47 (1.24—1.71)		1.38 (1.14—1.62	
** HGBd cut-point after PSW**	reference	< .001	reference	< .001	reference	< .001
≤ 18						
> 18	1.42 (1.17—1.72)		1.42 (1.17—1.72)		1.42 (1.17—1.74)	
**Patient without preoperative anemia**						
**Continous HGBd**	1.01 (1.01—1.02	< .001	1.01 (1.01—1.02)	< .001	1.01 (1.01—1.02)	< .001
**HGBd quintile, mmHg**						
< 5	reference		reference		reference	
5—12	1.52 (1.22—1.82)	< .001	1.49 (1.19—1.78)	< .001	1.52 (1.22—1.82)	< .001
12—18	1.67 (1.34—2.01)	< .001	1.64 (1.32—1.97)	< .001	1.68 (1.35—2.02)	< .001
18—26	1.60 (1.28—1.92)	< .001	1.56 (1.24—1.87)	< .001	1.61 (1.27—1.94)	< .001
> 26	1.92 (1.55—2.30)	< .001	1.83 (1.47—2.18)	< .001	1.86 (1.46—2.26)	< .001
**HGBd cut-point before PSW**	reference	< .001	reference	< .001	reference	< .001
≤ 43						
> 43	1.96 (1.52—2.41)		1.91 (1.48—2.35)		1.81 (1.35—2.27)	
**HGBd cut-point after PSW**	reference	< .001	reference	< .001	reference	< .001
≤ 43						
> 43	2.68 (1.76—4.07)		2.68 (1.76—4.08)		2.88 (1.85—4.50)	

Model 1: Crude model

Model 2: Crude model + age, gender

Full model: Model 2 + hypertension, preoperative albumin level, cancer surgery, intraperitoneal surgery, intraoperative blood transfusion, intraoperative hypotension, intraoperative dexmedetomidine and colloid use

*HGBd* Hemoglobin drop, the difference between preoperative mean hemoglobin and perioperative minimal hemoglobin, g/L

*aOR* adjusted odds ratio of AKI for hemoglobin drop

*CI* Confidence Interval

*PSW* Propensity Score Weighting

>

The fully adjusted odds ratio for patients with or without preoperative anemia was 1.38 (hemoglobin drop < 18 g/L as reference, 95%CI:1.14—1.62), *P* < 0.001; and 1.81 (hemoglobin drop < 43 g/L as reference, 95%CI:1.35—2.27), *P* < 0.001, respectively.

In the multivariate logistic regression, each variable included in the models demonstrated a variance inflation factor of no more than 3, suggesting no multicollinearity. Multivariate models with or without hemoglobin drop showed good discrimination [c-statistics were 0.697 and 0.714].

### Heterogeneity and sensitivity analysis

**Fig. 3:**
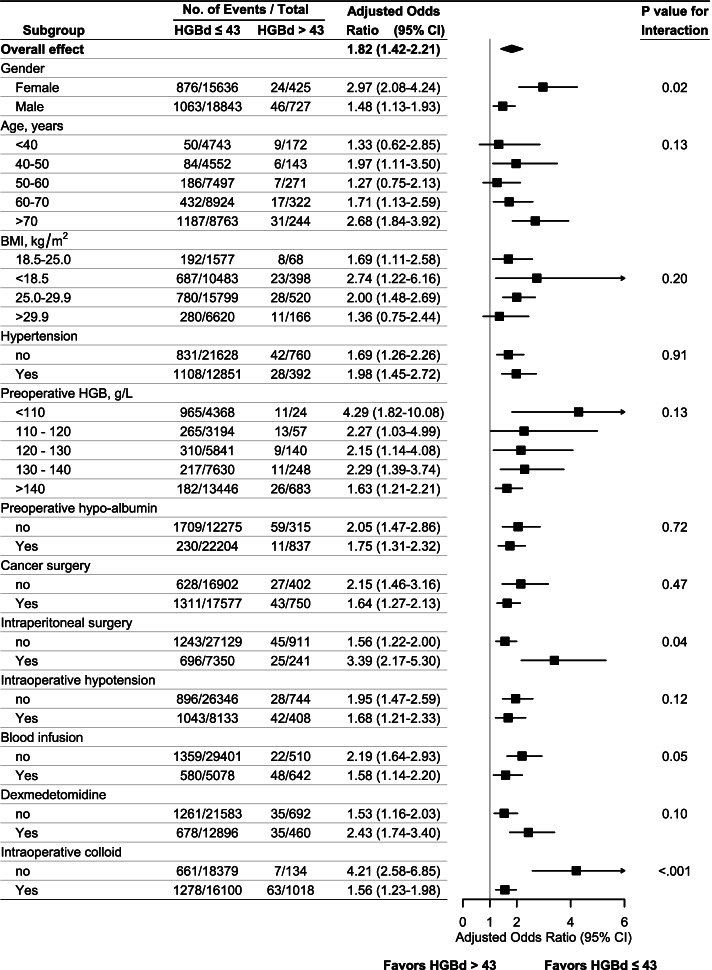
Subgroup analyses stratified by patient and operative variables. The adjusted covariates include age, gender, body mass index, hypertension, preoperative hemoglobin and albumin level, cancer surgery, intraperitoneal surgery, intraoperative blood transfusion, intraoperative hypotension, intraoperative dexmedetomidine, and colloid use. HGBd, hemoglobin drop, the difference between preoperative mean hemoglobin and minimal perioperative hemoglobin, g/L

An analysis of patients’ subgroups based on their characteristics showed interaction in gender, whether intraperitoneal surgery or intraoperative colloid infusion. Female patients undergoing intraperitoneal surgery with preoperative anemia were inclined to suffer AKI (Fig. 3, Supplementary Table S4, S5). The relationship between hemoglobin drop and AKI was qualitatively preserved across the sensitivity analyses (Supplementary Table S4-6). After a 1:1 match, the adjusted odds ratio was 1.94 (1.59 ~ 2.30), *P* < 0.001.

### Results of the analysis after the PS weighting

After the PSW, we divided the entire dataset into two groups, i.e., patients with hemoglobin drop ≤ or > 43 g/L. The balanced characteristics of the two cohorts were showed in Table S7. The recalculated logistic odds ratio for hemoglobin drop’s cut-off point (hemoglobin drop ≤ 43 mmHg as reference) was 3.29 (2.00—5.40), *P* < 0.001 (Table 3).

For stratified datasets, the adjusted PSW odds ratio for patients with or without preoperative anemia was 1.42 (hemoglobin drop < 18 g/L as reference, 95%CI:1.17—1.74), *P* < 0.001; and 2.88 (hemoglobin drop < 43 g/L as reference, 95%CI:1.85—4.50), *P* < 0.001, respectively (Table 3).

### Timeliness between hemoglobin and creatinine

This study found the timeliness between perioperative hemoglobin level and corresponding creatinine. Creatinine level changed simultaneously with hemoglobin level within five postoperative days (Supplementary figure S2, S3). After day 5, this phenomenon disappeared.

## Discussion

The present study showed that postoperative AKI occurred in 5.9% of adult patients undergoing elective noncardiac non-kidney surgery. Multivariable adjustment before or after PSW showed that higher hemoglobin drop was strongly associated with increased AKI occurrence. The perioperative hemoglobin drop thresholds were 18 and 43 g/L for patients with and without preoperative anemia. Hemoglobin drop thresholds for multi-mini-datasets with preoperative hemoglobin (span of 3 g/L) from 90 to 160 g/L demonstrated that, with the preoperative hemoglobin level decrease, the hemoglobin drop thresholds decrease dramatically accordingly.

The time window for acute kidney injury is seven days after surgery. However, previous studies have primarily focused on changes in hemoglobin levels within 24 h after surgery [41–43]. The changes in hemoglobin after 24 h have rarely been reported in published studies. Besides, intraoperative hemoglobin change is more severe. This study collected every possible data from the preoperative to the intraoperative and postoperative sessions to better observe the relation between hemoglobin drop and AKI.

It is accepted that preoperative hemoglobin level is a critical risk factor for postoperative AKI. Therefore, it is crucial to minimize preoperative hemoglobin level effects to understand the relation between hemoglobin drop and AKI. This study used stratification and multi-mini-datasets to accomplish this precondition.

In patients with preoperative anemia, various body reserves have already begun to mobilize or are even exhausted. Such patients are less tolerant of bleeding. More active intervention should be taken in these patients.

This study also discovered a same-day corresponding relationship between postoperative blood creatinine level (creatinine increase) and hemoglobin level (hemoglobin drop) (Figure S2, S3). As long as the hemoglobin level was reduced, an increase in the average blood creatinine level of the patients would be observed on the same day. This phenomenon was more evident in patients with AKI than in that without. This correspondence disappeared from the fifth day after surgery. The author speculates that most AKIs occurring within five days after surgery may be one of the reasons.

Due to clinical work characteristics, most postoperative patients have their blood routine and blood creatinine checked once a day. Although hemoglobin and blood creatinine was not obtained in the same test operation, the blood draw time was often the same, which indicates that the patient’s hemoglobin level and blood creatinine were likely to correspond within hours. Once kidney damage occurred, the dependence of this renal function on hemoglobin began to break away, and treatment was to no avail at this moment. It suggests that more frequent hemoglobin tests and earlier interventions may benefit patients with more AKI risk factors. This research proposes interesting directions for more in-depth research later. It also provides meaningful help for future clinical practice, as in the past, we made clinical decisions mainly on the postoperative hemoglobin level, ignoring the considerable influence of preoperative hemoglobin level on bleeding tolerance.

To the best of our knowledge, this is the first study focusing on the heterogeneity of hemoglobin drop thresholds for noncardiac non-kidney surgeries based on an analysis of real-world clinical data.

Our research has some advantages. After stratifying our analyses by perioperative variables, we found that our results remained reliable. Sensitivity analysis suggests that the relationship between hemoglobin drop and AKI does not change significantly depending on patient characteristics and the time frame of preoperative hemoglobin. We also proved the robustness of our conclusion by PSW analysis.

Although we tried our best to implement the best research methods and improve the database’s quality, various shortcomings and errors were inevitable. This dataset did not include proteinuria analysis, and preoperative chronic kidney disease and eGFR assessment were not entirely performed. The results of this study only draw associations and cannot imply causality. Thus, we cannot suggest that the management of achieving a hemoglobin drop below the thresholds will reduce AKI risk. Further randomized trials are needed. The single-center retrospective design might limit the generalizability of the present study, and external validation is warranted. Since each person’s hemoglobin level cannot be tested frequently, the lowest hemoglobin value in this study can only be equal to or higher than the patient’s actual lowest value. Therefore, the actual threshold may be higher than the threshold of this study.

## Conclusion

Heterogeneity of hemoglobin drop endurability exists after noncardiac non-kidney surgery. More care and earlier intervention should be put on female patients undergoing intraperitoneal surgery with preoperative anemia.

## Supplementary Information

**Additional file 1:** **Table S1.** Definitions of variables. **Table S2.** Postoperative events. **Table S3.** Improvement of Hemoglobin drop in full models for classification. **Table S4.** Sensitivity analyses. Multivariable logistic regression with surgery duration adjustment. **Table S5.** Sensitivity analyses. Multivariable logistic regression with exclusion of patients with Intraoperative hypotension. **Table S6.** Sensitivity analyses. Multivariable logistic regression with preoperative hemoglobin mean level within three months instead of the hemoglobin value tested closest to the date of surgery. **Table S7.** Patient characteristics and operative variables in cohorts by hemoglobin drop more or no more than 43 g/L after propensity score weighting.

**Additional file 2:** **Fig S1.** Restricted cubic spline function curves of the unadjusted and adjusted relationship between Hemoglobin drop and AKI probability. Shaded areas represent 95% confidence intervals.

**Additional file 3:** **Fig S2.** Timeliness between perioperative hemoglobin level and corresponding creatinine. The red line represented minimum hemoglobin level, with its corresponding red axis on the left. In plots A and B, the blue line represented creatinine level, with their corresponding blue axis on the right; in plots C and D, the blue line represented creatinine increment with their corresponding blue axis. Creatinine level changed simultaneously with hemoglobin level within five postoperative days. After day 5, this phenomenon disappeared.

**Additional file 4:** **Fig S3.** Timeliness between perioperative hemoglobin drop and corresponding creatinine. The red line represented maximum hemoglobin drop, with its corresponding red axis on the left. In plots A and B, the blue line represented creatinine level, with their corresponding blue axis on the right; in plots C and D, the blue line represented creatinine increment with their corresponding blue axis.

**Additional file 5:** **Fig S4.** Subgroup analyses stratified by patient and operative variables in patients without preoperative anemia.

**Additional file 6:** **Fig S5.** Subgroup analyses stratified by patient and operative variables in patients with preoperative anemia.

## Data Availability

The data that support the findings of this study are available from [Peking University First Hospital] but restrictions apply to the availability of these data, which were used under license for the current study, and so are not publicly available. Data are however available from the corresponding author upon reasonable request and with permission of [Peking University First Hospital]. The corresponding author can be contacted for requesting data.

## Abbreviations

ACE: Angiotensin-converting enzyme
CVD: Cardiovascular disease
SBP: Systolic blood pressure
DBP: Diastolic blood pressure
GAM: Generalized Additive Model
ICD: International classification of disease
AKI: Acute kidney injury
MJHSC: Modified John Hopkins hospital criteria
KDIGO: Kidney disease improving global outcomes
