# Supplementary material for: Threshold heterogeneity of perioperative hemoglobin drop for acute kidney injury after noncardiac surgery: a propensity score weighting analysis
Source: BMC Nephrol. 2022 Jun 11;23:206. doi: 10.1186/s12882-022-02834-3 (PMC9188693; doi:10.1186/s12882-022-02834-3)
Supplement: Supplementary file 1 — Additional file 1: Table S1. Definitions of variables. Table S2. Postoperative events. Table S3. Improvement of Hemoglobin drop in full models for classification. Table S4. Sensitivity analyses. Multivariable logistic regression with surgery duration adjustment. Table S5. Sensitivity analyses. Multivariable logistic regression with exclusion of patients with Intraoperative hypotension. Table S6. Sensitivity analyses. Multivariable logistic regression with preoperative hemoglobin mean level within three months instead of the hemoglobin value tested closest to the date of surgery. Table S7. Patient characteristics and operative variables in cohorts by hemoglobin drop more or no more than 43 g/L after propensity score weighting. [file 12882_2022_2834_MOESM1_ESM.docx]

Supplementary

**Table S1.** Definitions of variables.

| Variables | Definitions |
| --- | --- |
| Hemoglobin drop | AKA HGBd, Difference between preoperative hemoglobin level tested closest to the date of surgery and minimum intraoperative or postoperative hemoglobin level. |
| preoperative anemia | preoperative hemoglobin level less than 120 g/L in female or 130 g/L in male patients were defined as preoperative anemia. The hemoglobin was tested closest to the date of surgery. |
| Age | In years |
| Gender | Male or female |
| smoking | Patients’ smoking habit was collected by personal history in the database. |
| Body mass index | BMI, is a patient's weight in kilograms divided by the square of height in meters. |
| Hypertension | History of hypertension requiring anti-hypertensive medication recorded in the medical chart or Patients’ diagnosis with ICD code I10, I11, E10.722, E10.723, E11.722, E11.723, E14.722, E14.723, E16.8x101, E16.8x102, N28.917, O10.001, O10.101, O10.201, O10.301, O10.401, O10.901, O10.902, O11xx01, O13xx01, O16xx01, O99.415, P29.201, T70.202 were defined as hypertension. |
| Diabetes mellitus | A diagnosis of diabetes requiring oral medication or insulin or Patients’ diagnosis with ICD code E10, E11, E12, E13, E14 were defined as diabetes. |
| Heart insufficiency | Documented in the patient’s chart within 30 days before surgery. A diagnosis of congestive heart failure requiring diuretics or Patients’ diagnosis with ICD code I50, I97.104, T81.810, I11.001, I13.201, I97.106, N18.820, O29.102, O74.202, O75.402, O89.102, O99.408, O99.423. Alternatively, max preoperative BNP more than 400 pg/ml with signs and symptoms. |
| ASA-PS | 1 /2/3/4 or 5, recorded in the electronic database |
| Complexity of surgery | The modified John Hopkins Hospital criteria (MJHSC) were used for categorizing the surgical complexity. |
| Intraoperative blood loss | In mL, extracted from the electronic database |
| Intraoperative red blood cell transfusion | Administration of red blood cells or plasma during surgery |
| Intraoperative hypotension | intraoperative systolic blood pressure below 70 mmHg or diastolic blood pressure below 30 mmHg or mean blood pressure below 49 mmHg for more than or equal to 5 minutes, or mean blood pressure below decreases to more than 30% from baseline for more than or equal to 5 min. extracted and calculated from database. |
| Intraperitoneal operation | Operations intraperitoneally. Was defined by ICD codes. Such as lower esophagus, gastric, colon, appendix,rectus and bile and common duct surgeries. |
| Intraoperative mean HR | Intraoperative heart rate was collected and calculated by mean, and analyzed by IQR. |
| AKI | Acute kidney injury, and was defined by the patient’s postoperative serum creatinine increase to not less than 26.5 μmol/l within 48 hours, or 1.5 times from the baseline within 7 days after surgery, or initialization of blood dialysis. As the serum creatinine level fluctuates much postoperatively and could cause an inaccurate estimate of Glomerular Filtration Rate (eGFR), creatinine this study did not define AKI based on the GFR value or urine output. |
| Postoperative length of stay | Number of days from the surgery to discharge from the hospital |
| In-hospital mortality | Death during the hospital stay for the surgery |

ASA-PS, the American Society of Anaesthesiologists physical status;

ICD: The International Statistical Classification of Diseases;

Table S2. Postoperative events

| **Items** | **ALL (n=35631)** | **HGBd ≤ 43 (n=34479)** | **HGBd > 43 (n=1152)** | **P value** |
| --- | --- | --- | --- | --- |
| **AKI** | 2105(5.9%) | 1992(5.8%) | 113(9.8%) | <0.001 |
| **Grade 1** | 1846(6.0%) | 1276(5.1%) | 570(10.5%) | <0.001 |
| **Grade 2** | 138(0.4%) | 74(0.3%) | 64(1.2%) | <0.001 |
| **Grade 3** | 62(0.2%) | 37(0.1%) | 25(0.5%) | <0.001 |
| **ICU admission** | 2644(8.0%) | 1250(4.6%) | 1394(24.5%) | <0.001 |
| **Postoperative hospital stay (days)** | 5(3-9) | 5(2-8) | 8(5-13) | <0.001 |
| **In-hospital 7-day mortality** | 62(0.2%) | 30(0.1%) | 32(0.6%) | <0.001 |

Data are presented as n (%) or median (95% CI).

HGBd, hemoglobin drop, the difference between preoperative hemoglobin and perioperative minimal hemoglobin, g/L.

AKI: acute kidney injury.

ICU: intensive care unit

Table S3. Improvement of Hemoglobin drop in full models for classification.

|  | **AUC** | | **NRI** | | **IDI** | |
| --- | --- | --- | --- | --- | --- | --- |
|  | **Without HGBd** | **With HGBd** | **NRI (95% CI)** | **P-value** | **IDI (95% CI)** | **P-value** |
| **continous HGBd** | 0.697 | 0.714 | 0.2255 [0.1816 - 0.2694] | <.001 | 0.0076 [0.0061 - 0.0091] | <.001 |
| **HGBd quintile** |  | 0.702 | 0.0976 [0.0548 - 0.1404] | <.001 | 0.0016 [0.0010 - 0.0023] | <.001 |
| **HGBd cut point** |  | 0.700 | 0.0852 [0.0452 - 0.1253] | <.001 | 0.0009 [0.0004 - 0.0014] | 0.001 |

AUC: area under curve.

HGBd, hemoglobin drop, the difference between preoperative hemoglobin and perioperative minimal hemoglobin, g/L.

NRI: Net reclassification improvement

IDI: integrated discrimination improvement

Table S4. Sensitivity analyses. Multivariable logistic regression with surgery duration adjustment.

|  | **Patient/operative variables only** | | **Patient/operative variables and HGBd** | |
| --- | --- | --- | --- | --- |
|  | **aOR (95% CI)** | **P-value** | **aOR (95% CI)** | **P-value** |
| **Gender**, (female) | 1.32 (1.18 - 1.46) | <.001 | 1.32 (1.19 - 1.46) | <.001 |
| **Age**, (< 40 yr) | reference | <.001 | reference | <.001 |
| 40 - 50 | 1.45 (1.14 - 1.77) |  | 1.47 (1.15 - 1.79) |  |
| 50 - 60 | 1.56 (1.25 - 1.87) |  | 1.57 (1.25 - 1.89) |  |
| 60 - 70 | 1.35 (1.08 - 1.62) |  | 1.36 (1.09 - 1.63) |  |
| > 70 | 1.89 (1.52 - 2.26) |  | 1.91 (1.53 - 2.28) |  |
| **Body mass index**, (18.5-24.9 kg/m^2^) | reference | <.001 | reference | <.001 |
| <18.5 | 1.45 (1.16 - 1.73) |  | 1.45 (1.16 - 1.74) |  |
| 25.0-29.9 | 1.12 (0.99 - 1.24) |  | 1.12 (1.00 - 1.24) |  |
| >30.0 | 1.29 (1.12 - 1.47) |  | 1.31 (1.13 - 1.48) |  |
| **hypertension** | 0.99 (0.85 - 1.13) | 0.873 | 1.00 (0.86 - 1.14) | 0.986 |
| **Preoperative hemoglobin level,** 130 – 140 g/L | reference | <.001 | reference | <.001 |
| < 110 | 2.03 (1.72 - 2.34) |  | 2.06 (1.74 - 2.38) |  |
| 110 - 120 | 1.24 (1.02 - 1.47) |  | 1.26 (1.03 - 1.48) |  |
| 120 - 130 | 0.98 (0.82 - 1.14) |  | 0.99 (0.82 - 1.15) |  |
| > 140 | 1.26 (1.09 - 1.43) |  | 1.25 (1.08 - 1.42) |  |
| **Preoperative albumin level,** >40 mg/L | 1.76 (1.58 - 1.94) | <.001 | 1.77 (1.59 - 1.95) | <.001 |
| **Cancer to benign surgery** | 1.47 (1.32 - 1.63) | <.001 | 1.45 (1.30 - 1.61) | <.001 |
| **Intraperitoneal surgery** | 0.75 (0.66 - 0.83) | <.001 | 0.75 (0.67 - 0.84) | <.001 |
| **Intraoperative hypotension** | 1.74 (1.48 - 2.01) | <.001 | 1.72 (1.46 - 1.98) | <.001 |
| **Intraoperative blood transfusion** | 0.98 (0.86 - 1.10) | 0.780 | 0.92 (0.80 - 1.04) | 0.196 |
| **Intraoperative dexmedetomidine use** | 0.81 (0.73 - 0.89) | <.001 | 0.81 (0.73 - 0.89) | <.001 |
| **Intraoperative colloid use** | 1.24 (1.10 - 1.37) | <.001 | 1.21 (1.08 - 1.34) | 0.001 |
| **Surgery duration**, <120 min | reference |  | reference |  |
| 120 - 240 | 0.99 (0.87 - 1.11) |  | 1.00 (0.88 - 1.12) |  |
| 240 - 480 | 0.84 (0.72 - 0.96) |  | 0.83 (0.70 - 0.95) |  |
| >480 | 1.35 (0.99 - 1.70) |  | 1.27 (0.94 - 1.61) |  |
| **Hemoglobin drop^c^**, (≤ 43 g/L) | reference | - | reference | <.001 |
| > 43 | - |  | 1.67 (1.33 - 2.02) |  |

GA, general anesthesia

HGBd, hemoglobin drop, the difference between preoperative mean hemoglobin and perioperative minimal hemoglobin, g/L.

CI, confidence interval

aOR, adjusted odds ratio

Table S5. Sensitivity analyses. Multivariable logistic regression with exclusion of patients with Intraoperative hypotension.

|  | **Patient/operative variables only** | | **Patient/operative variables and HGBd** | |
| --- | --- | --- | --- | --- |
|  | **aOR (95% CI)** | **P-value** | **aOR (95% CI)** | **P-value** |
| **Gender**, (female) | 1.31 (1.13 - 1.49) | <.001 | 1.32 (1.14 - 1.50) | <.001 |
| **Age**, (< 40 yr) | reference | <.001 | reference | <.001 |
| 40 - 50 | 1.49 (1.14 - 1.85) |  | 1.50 (1.14 - 1.87) |  |
| 50 - 60 | 1.52 (1.18 - 1.87) |  | 1.54 (1.19 - 1.88) |  |
| 60 - 70 | 1.36 (1.05 - 1.67) |  | 1.37 (1.06 - 1.69) |  |
| > 70 | 2.01 (1.57 - 2.45) |  | 2.02 (1.58 - 2.46) |  |
| **Body mass index**, (18.5-24.9 kg/m^2^) | reference | <.001 | reference | <.001 |
| <18.5 | 1.42 (1.09 - 1.76) |  | 1.41 (1.08 - 1.74) |  |
| 25.0-29.9 | 1.08 (0.93 - 1.23) |  | 1.09 (0.94 - 1.24) |  |
| >30.0 | 1.31 (1.08 - 1.53) |  | 1.33 (1.10 - 1.56) |  |
| **hypertension** | 1.04 (0.87 - 1.20) | 0.673 | 1.04 (0.87 - 1.20) | 0.663 |
| **Preoperative hemoglobin level,** 130 – 140 g/L | reference | <.001 | reference | <.001 |
| < 110 | 2.22 (1.77 - 2.67) |  | 2.32 (1.85 - 2.79) |  |
| 110 - 120 | 1.25 (0.95 - 1.56) |  | 1.28 (0.97 - 1.59) |  |
| 120 - 130 | 1.05 (0.82 - 1.27) |  | 1.06 (0.83 - 1.28) |  |
| > 140 | 1.41 (1.16 - 1.66) |  | 1.39 (1.14 - 1.64) |  |
| **Preoperative albumin level,** >40 mg/L | 1.73 (1.51 - 1.95) | <.001 | 1.75 (1.53 - 1.97) | <.001 |
| **Cancer to benign surgery** | 1.62 (1.41 - 1.82) | <.001 | 1.59 (1.39 - 1.80) | <.001 |
| **Intraperitoneal surgery** | 0.78 (0.66 - 0.90) | 0.001 | 0.78 (0.66 - 0.90) | 0.001 |
| **Intraoperative hypotension** | - | - | - | - |
| **Intraoperative blood transfusion** | 1.02 (0.86 - 1.19) | 0.781 | 0.96 (0.80 - 1.12) | 0.630 |
| **Intraoperative dexmedetomidine use** | 0.81 (0.71 - 0.91) | 0.001 | 0.80 (0.70 - 0.90) | 0.001 |
| **Intraoperative colloid use** | 1.36 (1.19 - 1.53) | <.001 | 1.32 (1.15 - 1.49) | <.001 |
| **Hemoglobin drop^c^**, (≤ 43 g/L) | reference | - | reference | <.001 |
| > 43 | - |  | 1.95 (1.39 - 2.52) |  |

GA, general anesthesia

HGBd, hemoglobin drop, the difference between preoperative mean hemoglobin and perioperative minimal hemoglobin, g/L.

CI, confidence interval

aOR, adjusted odds ratio

Table S6. Sensitivity analyses. Multivariable logistic regression with preoperative hemoglobin mean level within three months instead of the hemoglobin value tested closest to the date of surgery.

|  | **Patient/operative variables only** | | **Patient/operative variables and HGBd** | |
| --- | --- | --- | --- | --- |
|  | **aOR (95% CI)** | **P-value** | **aOR (95% CI)** | **P-value** |
| **Gender**, (female) | 1.32 (1.18 - 1.45) | <.001 | 1.31 (1.18 - 1.45) | <.001 |
| **Age**, (< 40 yr) | reference | <.001 | reference | <.001 |
| 40 - 50 | 1.45 (1.13 - 1.77) |  | 1.46 (1.14 - 1.78) |  |
| 50 - 60 | 1.55 (1.24 - 1.86) |  | 1.56 (1.24 - 1.87) |  |
| 60 - 70 | 1.34 (1.07 - 1.61) |  | 1.35 (1.08 - 1.62) |  |
| > 70 | 1.87 (1.51 - 2.24) |  | 1.89 (1.52 - 2.26) |  |
| **Body mass index**, (18.5-24.9 kg/m^2^) | reference | <.001 | reference | <.001 |
| <18.5 | 1.45 (1.16 - 1.73) |  | 1.45 (1.16 - 1.73) |  |
| 25.0-29.9 | 1.11 (0.99 - 1.23) |  | 1.12 (0.99 - 1.24) |  |
| >30.0 | 1.29 (1.11 - 1.46) |  | 1.30 (1.13 - 1.48) |  |
| **hypertension** | 1.00 (0.85 - 1.14) | 0.961 | 1.01 (0.87 - 1.15) | 0.903 |
| **Preoperative hemoglobin level,** 130 – 140 g/L | reference | <.001 | reference | <.001 |
| < 110 | 2.04 (1.72 - 2.35) |  | 2.07 (1.75 - 2.39) |  |
| 110 - 120 | 1.24 (1.02 - 1.47) |  | 1.25 (1.02 - 1.48) |  |
| 120 - 130 | 0.98 (0.82 - 1.14) |  | 0.98 (0.82 - 1.15) |  |
| > 140 | 1.26 (1.09 - 1.44) |  | 1.25 (1.08 - 1.42) |  |
| **Preoperative albumin level,** >40 mg/L | 1.76 (1.58 - 1.94) | <.001 | 1.76 (1.58 - 1.94) | <.001 |
| **Cancer to benign surgery** | 1.45 (1.30 - 1.59) | <.001 | 1.43 (1.28 - 1.57) | <.001 |
| **Intraperitoneal surgery** | 0.74 (0.65 - 0.82) | <.001 | 0.75 (0.66 - 0.83) | <.001 |
| **Intraoperative hypotension** | 1.73 (1.47 - 1.98) | <.001 | 1.70 (1.45 - 1.94) | <.001 |
| **Intraoperative blood transfusion** | 0.98 (0.86 - 1.09) | 0.699 | 0.91 (0.80 - 1.03) | 0.149 |
| **Intraoperative dexmedetomidine use** | 0.81 (0.73 - 0.89) | <.001 | 0.81 (0.73 - 0.88) | <.001 |
| **Intraoperative colloid use** | 1.21 (1.09 - 1.34) | <.001 | 1.18 (1.06 - 1.31) | 0.001 |
| **Hemoglobin drop^c^**, (≤ 43 g/L) | reference | - | reference | <.001 |
| > 43 | - |  | 1.66 (1.33 - 2.00) |  |

GA, general anesthesia

HGBd, hemoglobin drop, the difference between preoperative mean hemoglobin and perioperative minimal hemoglobin, g/L.

CI, confidence interval

aOR, adjusted odds ratio

Table S7. Patient characteristics and operative variables in cohorts by hemoglobin drop more or no more than 43 g/L after propensity score weighting.

| **characteristic** | **HGBd** ≤ **43** (n= 35552.1) | **HGBd > 43** (n= 27917.4) | **P value** | **SMD**, % |
| --- | --- | --- | --- | --- |
| **Age** [yr; median (IQR)] | 61 [49-71] | 62 [49-70] | 0.905 | 0.7 |
| **Male gender**, [n (%)] | 19533.1(54.9%) | 16448.3(58.9%) | 0.223 | 8.0 |
| **Body mass index**, [mean (SD)] kg/m^2^ | 24.47 (3.80) | 24.42 (3.78) | 0.842 | 1.4 |
| **smoking** | 5287.7 (14.9%) | 4599.4 (16.5%) | 0.493 | 4.4 |
| **drinking** | 4524.6 (12.7%) | 4481.4 (16.1%) | 0.142 | 9.5 |
| **Co-existing disease** |  |  |  |  |
| hypertension | 13214.2(37.2%) | 9900.1(35.5%) | 0.581 | 3.5 |
| Coronary artery disease | 5861.6(16.5%) | 3881.6(13.9%) | 0.237 | 7.2 |
| Heart failure | 534.7(1.5%) | 242.4(0.9%) | 0.135 | 5.9 |
| Arrhythmia | 1237.3(3.5%) | 1582(5.7%) | 0.094 | 10.5 |
| Peripheral arterial disease | 678.9(1.9%) | 229.1(0.8%) | 0.092 | 9.4 |
| Stroke | 3514.6(9.9%) | 2639.8(9.5%) | 0.841 | 1.5 |
| diabetes mellitus | 5133.4 (14.4%) | 3680.2 (13.2%) | 0.596 | 3.6 |
| **Preoperative serum creatinine**, [mean (SD)] mmol/L | 81.07 (19.24) | 81.55 (17.90) | 0.649 | 2.6 |
| **Preoperative serum albumin**, [mean (SD)] g/L | 40.97 (5.88) | 41.37 (6.63) | 0.426 | 6.3 |
| **rCRI** |  |  | 0.066 | 16 |
| 0 | 30229.8(85%) | 24657.2(88.3%) |  |  |
| 1 | 3225.8(9.1%) | 1662.4(6%) |  |  |
| 2 | 1462.6(4.1%) | 1402.3(5%) |  |  |
| ≥ 3 | 633.9(1.8%) | 195.5(0.7%) |  |  |
| **ASA** |  |  | 0.775 | 5.2 |
| I | 5848.3 (16.4%) | 4227.7 (15.1%) |  |  |
| II | 25568.2 (71.9%) | 20225.4 (72.4%) |  |  |
| III | 3974.4 (11.2%) | 3386.5 (12.1%) |  |  |
| IV or V | 161.2 (0.5%) | 77.7 (0.3%) |  |  |
| **Surgery type** |  |  | 0.097 | 25 |
| Eye/ear/throat | 283.5(0.8%) | 166.9(0.6%) |  |  |
| Integumentary | 319.3(0.9%) | 279.6(1%) |  |  |
| Genital/urinary | 8566(24.1%) | 8864.7(31.8%) |  |  |
| Musculoskeletal | 4452.4(12.5%) | 2575(9.2%) |  |  |
| Nervous | 2535.1(7.1%) | 1909.6(6.8%) |  |  |
| Vascular | 745.4(2.1%) | 940(3.4%) |  |  |
| Digestive | 13990.7(39.4%) | 10802.2(38.7%) |  |  |
| Respiratory | 3417.3(9.6%) | 1951.6(7%) |  |  |
| Other | 1242.3(3.5%) | 427.6(1.5%) |  |  |
| **Intraperitoneal surgery** | 7579.4 (21.3%) | 5950.0 (21.3%) | 0.998 | < 0.1 |
| **Cancer surgery** | 18261.2 (51.4%) | 14765.6 (52.9%) | 0.644 | 3.1 |
| **Surgery time**, [min; median (IQR)] | 129 [74-214] | 133 [79-215] | 0.145 | 0.2 |
| **Anesthesia duration**, [min; median (IQR)] | 207 [140-306] | 220 [151-323] | 0.053 | 11.4 |
| **Anesthesia type** |  |  | 0.269 | 8.6 |
| General anesthesia | 31007.5 (87.2%) | 25107.7 (89.9%) |  |  |
| General anesthesia + epidural/nerve block | 4544.6 (12.8%) | 2809.7 (10.1%) |  |  |
| **Intraoperative ﬂuid administration** [median (IQR)] |  |  |  |  |
| Infusion volume | 1600 [1100- 2450] | 2050 [1200- 2900] | <.001 | 26.2 |
| Crystal | 1300 [1000-1900] | 1600 [1100-2300] | <.001 | 32.2 |
| Colloid | 0 [0-500] | 500 [0-500] | 0.151 | 8.2 |
| **Estimated blood loss**, [ml; median (IQR)] | 50 [0-200] | 100 [0-400] | <.001 | 22 |
| **Intraoperative blood infusion** | 5639.8(15.9%) | 4760.1(17.1%) | 0.471 | 3.2 |
| **Urine**, [ml; median (IQR)] | 200 [0-500] | 200 [0-550] | 0.660 | 4.4 |
| **Intraoperative hypotension** | 8452.1(23.8%) | 7519.4(26.9%) | 0.206 | 7.3 |
| **Intraoperative mean HR**, bpm |  |  | 0.904 | 4.9 |
| 60-65 | 7525.7(21.5%) | 5600.5(20.2%) |  |  |
| <60 | 9720.7(27.8%) | 7439.6(26.8%) |  |  |
| 65-75 | 10328.2(29.5%) | 8677.6(31.2%) |  |  |
| >75 | 7413(21.2%) | 6064.5(21.8%) |  |  |

SMD: standard mean difference, less than 15% is considered well balanced.

rCRI: revised cardiac risk index

HGBd, hemoglobin drop, the difference between preoperative mean hemoglobin and perioperative minimal hemoglobin, g/L.

**Figure legends**

Fig S1. Restricted cubic spline function curves of the unadjusted and adjusted relationship between Hemoglobin drop and AKI probability. Shaded areas represent 95% conﬁdence intervals.

AKI: acute kidney injury

Fig S2. Timeliness between perioperative hemoglobin level and corresponding creatinine. The red line represented minimum hemoglobin level, with its corresponding red axis on the left. In plots A and B, the blue line represented creatinine level, with their corresponding blue axis on the right; in plots C and D, the blue line represented creatinine increment with their corresponding blue axis. Creatinine level changed simultaneously with hemoglobin level within five postoperative days. After day 5, this phenomenon disappeared.

AKI: acute kidney injury

Fig S3. Timeliness between perioperative hemoglobin drop and corresponding creatinine. The red line represented maximum hemoglobin drop, with its corresponding red axis on the left. In plots A and B, the blue line represented creatinine level, with their corresponding blue axis on the right; in plots C and D, the blue line represented creatinine increment with their corresponding blue axis.

AKI: acute kidney injury

Fig S4. Subgroup analyses stratiﬁed by patient and operative variables in patients without preoperative anemia.

The adjusted covariates include age, gender, body mass index, hypertension, preoperative albumin level, cancer surgery, intraperitoneal surgery, intraoperative blood transfusion, intraoperative hypotension, intraoperative dexmedetomidine, and colloid use.

HGBd, hemoglobin drop, the difference between preoperative mean hemoglobin and minimal perioperative hemoglobin, g/L.

Fig S5. Subgroup analyses stratiﬁed by patient and operative variables in patients with preoperative anemia.

The adjusted covariates include age, gender, body mass index, hypertension, preoperative albumin level, cancer surgery, intraperitoneal surgery, intraoperative blood transfusion, intraoperative hypotension, intraoperative dexmedetomidine, and colloid use.

HGBd, hemoglobin drop, the difference between preoperative mean hemoglobin and minimal perioperative hemoglobin, g/L.
