# Supplementary figures and images for: Threshold heterogeneity of perioperative hemoglobin drop for acute kidney injury after noncardiac surgery: a propensity score weighting analysis
Source: BMC Nephrol. 2022 Jun 11;23:206. doi: 10.1186/s12882-022-02834-3 (PMC9188693; doi:10.1186/s12882-022-02834-3)

**Crude Model**

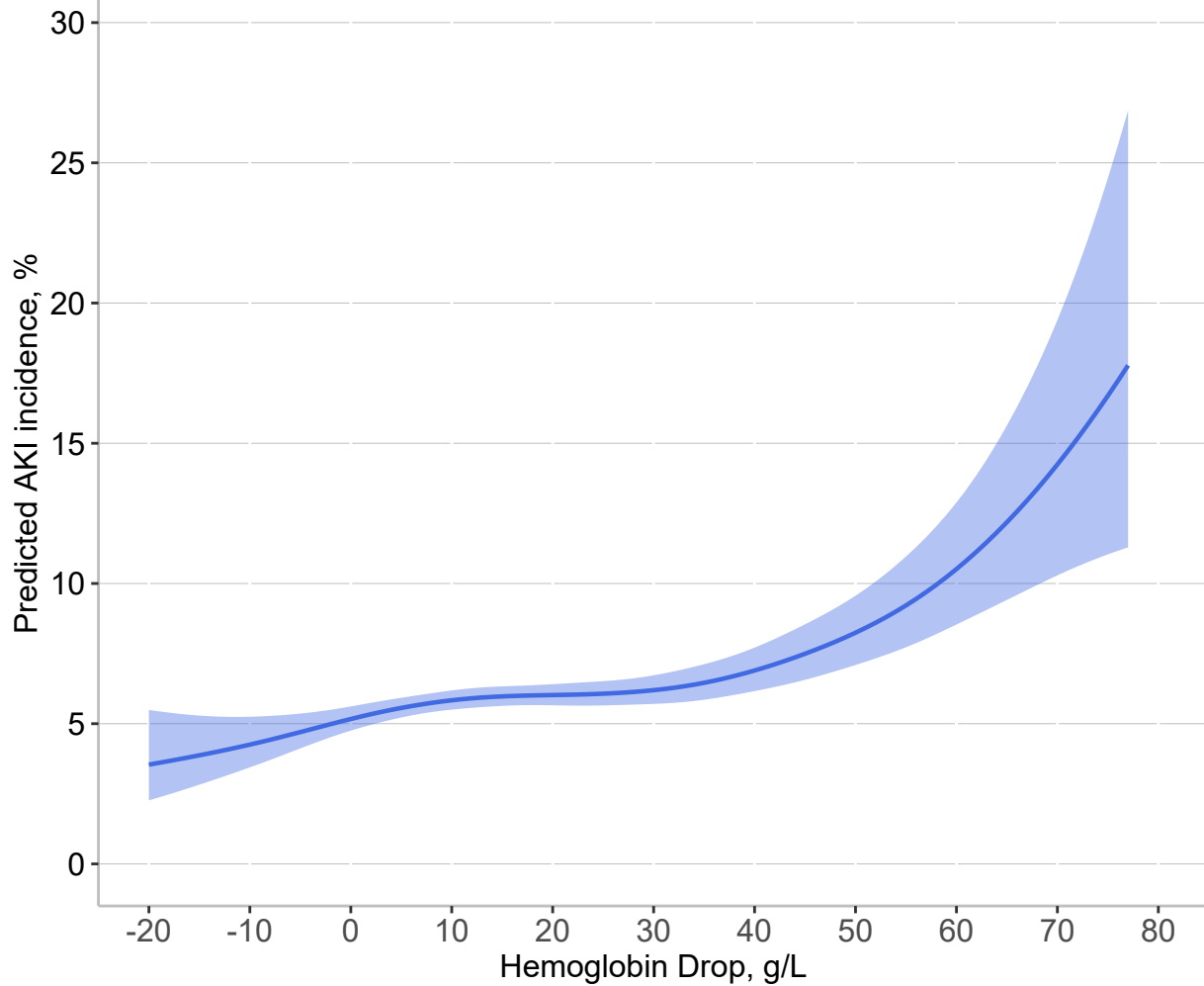

**Adjusted Model**

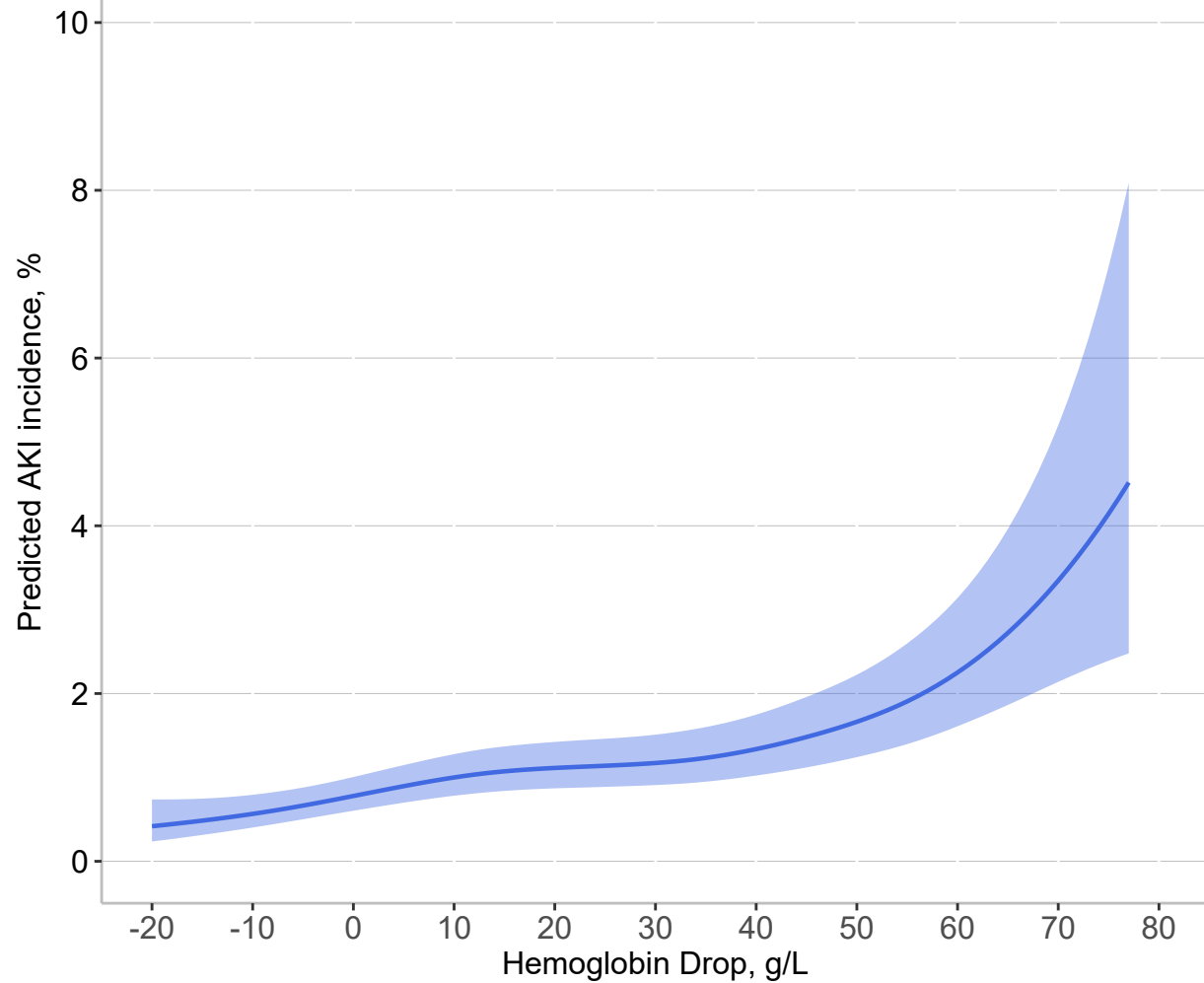

Supplement: Supplementary file 2 — Additional file 2: Fig S1. Restricted cubic spline function curves of the unadjusted and adjusted relationship between Hemoglobin drop and AKI probability. Shaded areas represent 95% confidence intervals. [file 12882_2022_2834_MOESM2_ESM.pdf]

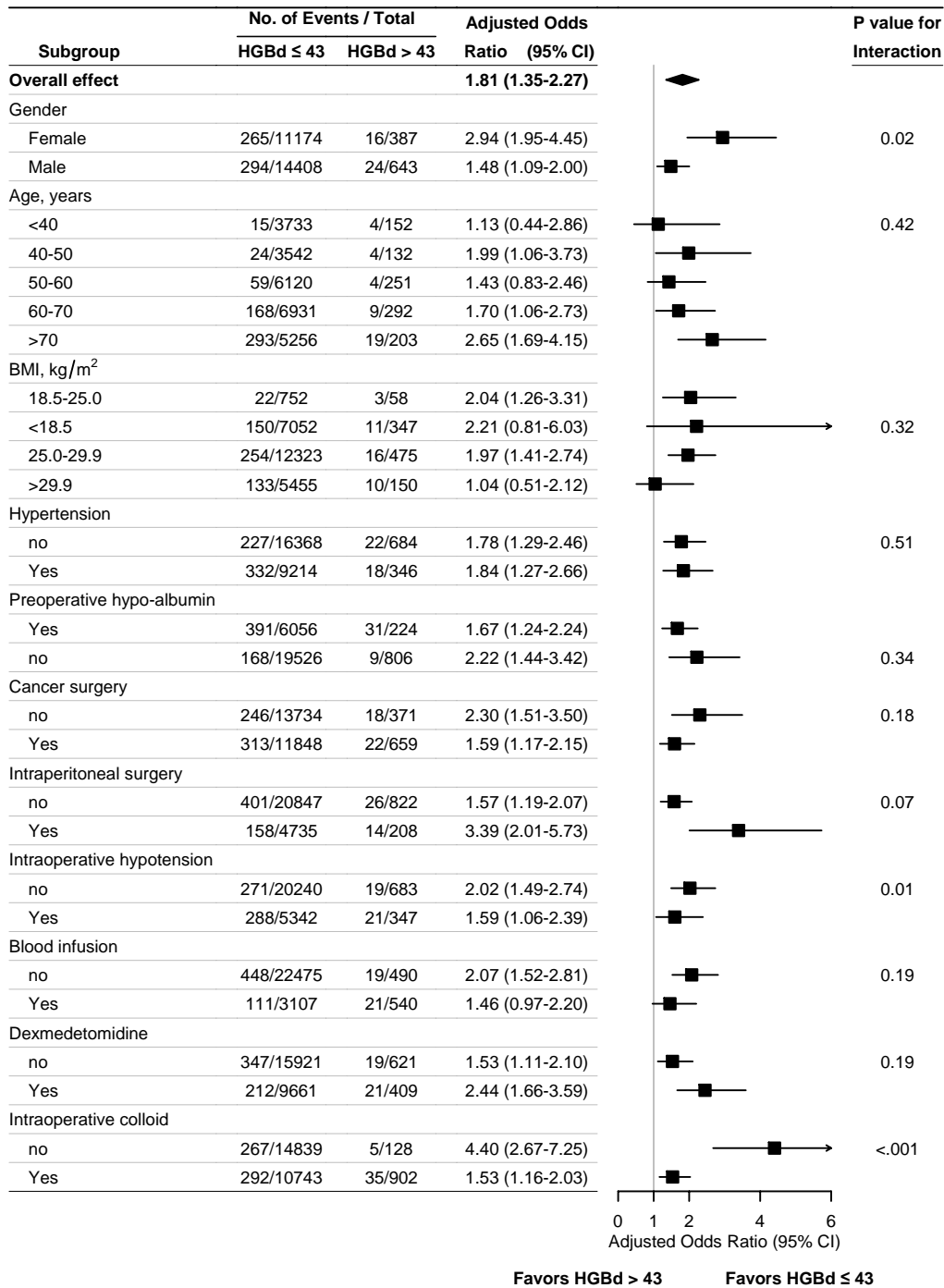

Supplement: Supplementary file 5 — Additional file 5: Fig S4. Subgroup analyses stratified by patient and operative variables in patients without preoperative anemia. [file 12882_2022_2834_MOESM5_ESM.pdf]

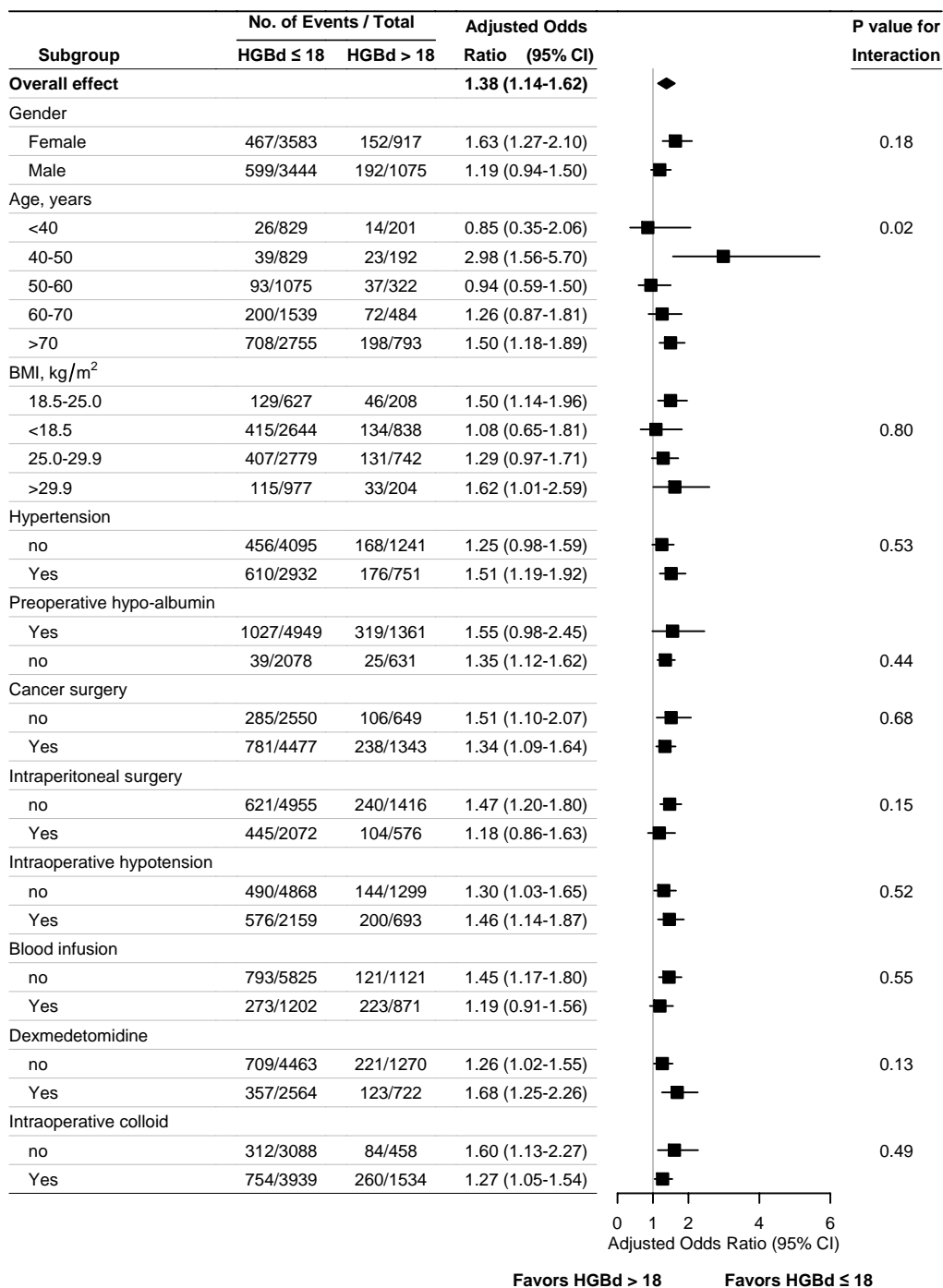

Supplement: Supplementary file 6 — Additional file 6: Fig S5. Subgroup analyses stratified by patient and operative variables in patients with preoperative anemia. [file 12882_2022_2834_MOESM6_ESM.pdf]
