# Supplementary material for: Threshold heterogeneity of perioperative hemoglobin drop for acute kidney injury after noncardiac surgery: a propensity score weighting analysis
Source: BMC Nephrol. 2022 Jun 11;23:206. doi: 10.1186/s12882-022-02834-3 (PMC9188693; doi:10.1186/s12882-022-02834-3)
Supplement: Supplementary file 3 — Additional file 3: Fig S2. Timeliness between perioperative hemoglobin level and corresponding creatinine. The red line represented minimum hemoglobin level, with its corresponding red axis on the left. In plots A and B, the blue line represented creatinine level, with their corresponding blue axis on the right; in plots C and D, the blue line represented creatinine increment with their corresponding blue axis. Creatinine level changed simultaneously with hemoglobin level within five postoperative days. After day 5, this phenomenon disappeared. [file 12882_2022_2834_MOESM3_ESM.pdf]

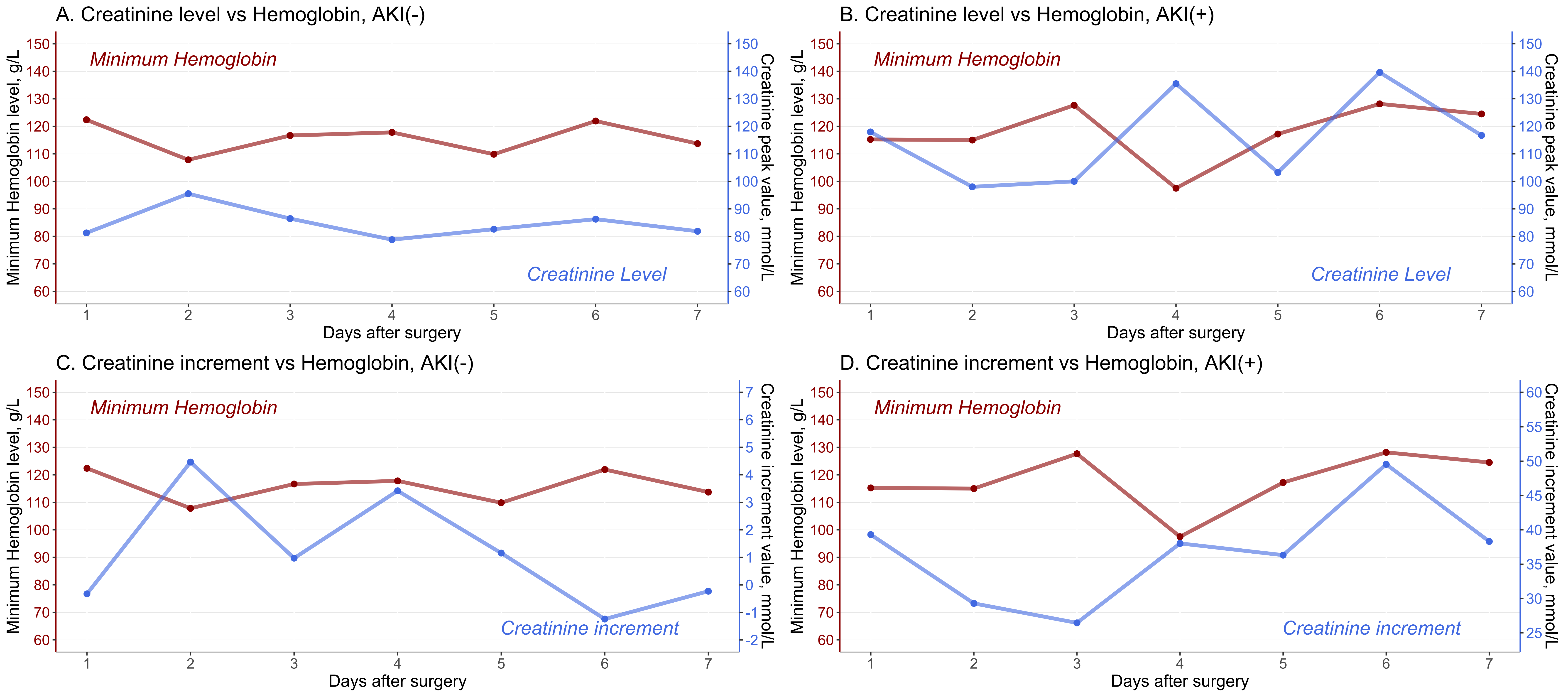
A. Creatinine level vs Hemoglobin, AKI(-)
B. Creatinine level vs Hemoglobin, AKI(+)
C. Creatinine increment vs Hemoglobin, AKI(-)
D. Creatinine increment vs Hemoglobin, AKI(+)
Minimum Hemoglobin
Creatinine Level
Creatinine increment
Minimum Hemoglobin level, g/L
Creatinine peak value, mmol/L
Creatinine increment value, mmol/L
Days after surgery
150
140
130
120
110
100
90
80
70
60
7
6
5
4
3
2
1
0
-1
-2
55
50
45
40
35
30
25
