# Supplementary material for: Threshold heterogeneity of perioperative hemoglobin drop for acute kidney injury after noncardiac surgery: a propensity score weighting analysis
Source: BMC Nephrol. 2022 Jun 11;23:206. doi: 10.1186/s12882-022-02834-3 (PMC9188693; doi:10.1186/s12882-022-02834-3)
Supplement: Supplementary file 4 — Additional file 4: Fig S3. Timeliness between perioperative hemoglobin drop and corresponding creatinine. The red line represented maximum hemoglobin drop, with its corresponding red axis on the left. In plots A and B, the blue line represented creatinine level, with their corresponding blue axis on the right; in plots C and D, the blue line represented creatinine increment with their corresponding blue axis. [file 12882_2022_2834_MOESM4_ESM.pdf]

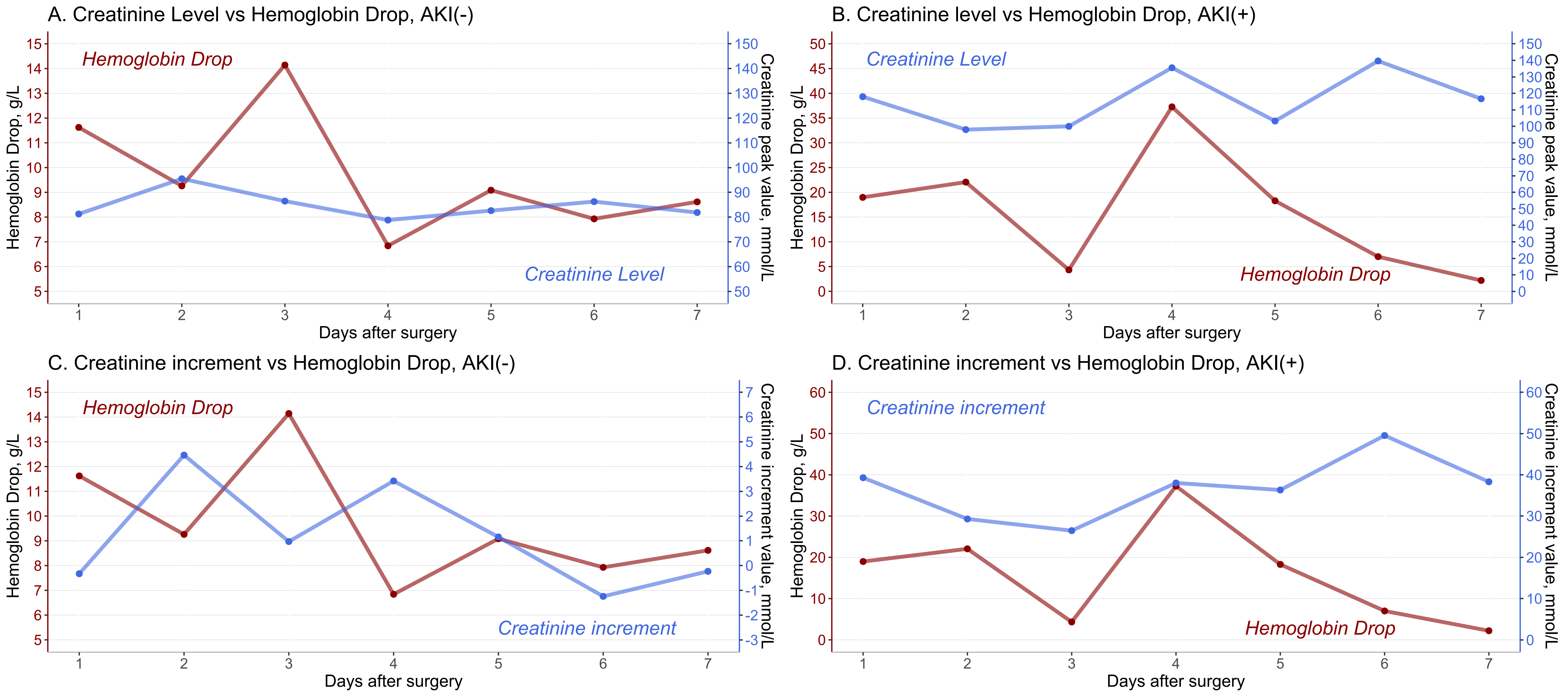

A. Creatinine Level vs Hemoglobin Drop, AKI(-)
Hemoglobin Drop
Creatinine Level
Hemoglobin Drop, g/L
Creatinine peak value, mmol/L
Days after surgery
B. Creatinine level vs Hemoglobin Drop, AKI(+)
Creatinine Level
Hemoglobin Drop
Hemoglobin Drop, g/L
Creatinine peak value, mmol/L
Days after surgery
C. Creatinine increment vs Hemoglobin Drop, AKI(-)
Hemoglobin Drop
Creatinine increment
Hemoglobin Drop, g/L
Creatinine increment value, mmol/L
Days after surgery
D. Creatinine increment vs Hemoglobin Drop, AKI(+)
Creatinine increment
Hemoglobin Drop
Hemoglobin Drop, g/L
Creatinine increment value, mmol/L
Days after surgery
